# Rice *Premature Leaf Senescence 2*, Encoding a Glycosyltransferase (GT), Is Involved in Leaf Senescence

**DOI:** 10.3389/fpls.2018.00560

**Published:** 2018-04-26

**Authors:** Min Wang, Tao Zhang, Hao Peng, Sheng Luo, Juejie Tan, Kaifeng Jiang, Yueqin Heng, Xin Zhang, Xiuping Guo, Jiakui Zheng, Zhijun Cheng

**Affiliations:** ^1^National Key Facility for Crop Gene Resources and Genetic Improvement, Institute of Crop Science, Chinese Academy of Agricultural Sciences, Beijing, China; ^2^Institute of Rice and Sorghum, Sichuan Academy of Agricultural Sciences, Deyang, China; ^3^Department of Life Science and Engineering, Jining University, Jining, China

**Keywords:** glycosyltransferase, GT, leaf senescence, *Oryza sativa*, *PLS2*, sucrose

## Abstract

Premature leaf senescence (PLS), which has a significant impact on yield, is caused by various underlying mechanisms. Glycosyltransferases, which function in glycosyl transfer from activated nucleotides to aglycones, are involved in diverse biological processes, but their roles in rice leaf senescence remain elusive. Here, we isolated and characterized a leaf senescence-related gene from the *Premature Leaf Senescent* mutant (*pls2*). The mutant phenotype began with leaf yellowing at tillering and resulted in PLS during the reproductive stage. Leaf senescence was associated with an increase in hydrogen peroxide (H_2_O_2_) content accompanied with pronounced decreases in net photosynthetic rate, stomatal conductance, and transpiration rate. Map-based cloning revealed that a mutation in LOC_Os03g15840 (*PLS2*), a putative glycosyltransferase- encoding gene, was responsible for the defective phenotype. *PLS2* expression was detected in all tissues surveyed, but predominantly in leaf mesophyll cells. Subcellular localization of the PLS2 was in the endoplasmic reticulum. The *pls2* mutant accumulated higher levels of sucrose together with decreased expression of sucrose metabolizing genes compared with wild type. These data suggested that the *PLS2* allele is essential for normal leaf senescence and its mutation resulted in PLS.

## Introduction

Plant senescence is an age-dependent behavior in plant development under normal growth condition (Lee et al., 2001; Woo et al., 2001). During senescence, leaf cells undergo dramatic metabolic changes, including chlorophyll breakdown and hydrolysis of macromolecules (lipids, proteins, and nucleic acids), that results in leaf cell death (Kong et al., 2006; Schippers et al., 2015). In agriculture, delayed leaf senescence (stay green) provides opportunities to prolong photosynthetic capacity and increase crop yield (Thomas and Howarth, 2000). PLS is triggered by various external factors (such as drought, salinity, shading, or biotic stress) as well as physiological factors such as endogenous sugar content, or plant hormone levels (van Doorn, 2008; Schippers et al., 2015; Abdelrahman et al., 2017), and usually causes yield loss (Bai et al., 2015; Rao et al., 2015). Therefore, an in-depth understanding of the molecular mechanism of leaf senescence is important in delaying leaf senescence and increasing cereal crop production (Wu et al., 2012; Gregersen et al., 2013).

By now, many research advances of leaf senescence at the molecular level have been achieved through the isolation and characterization of dozens of senescence-related mutants and senescence-associated genes (*SAGs*) (Buchanan-Wollaston et al., 2003; Lim et al., 2007; Li et al., 2012). These *SAGs* are usually involved in various biological processes, such as the breakdown of chlorophyll, degradation of chloroplasts, plant hormone synthesis and signaling, and biotic and abiotic stress responses (Kong et al., 2006; Jiao et al., 2012; Li et al., 2012; Liang et al., 2014; Sakuraba et al., 2015). Several *SAGs* in rice have been isolated and functionally characterized. For example, the NB-domain-containing protein encoding gene, *RLS1* (*rapid leaf senescence 1*), is involved in an autophagy-like process of chloroplast degradation (Jiao et al., 2012). The highly increased transcription level of *OsABC1-2* (an Abc1 kinase family gene), a chloroplast membrane-localized kinase encoding gene, is dramatically suppressed by dark treatment and its over-expression improves plant resistance in extended periods of darkness (Gao et al., 2012). *OsNAP* (rice NAC-like, activated by apetala3/pistillata) exerts roles in regulating expressions of an age-dependent manner *SAGs* and ABA biosynthesis related genes (Liang et al., 2014). Overexpressing rice *OsWRKY42* (one transcription factor of WRKY family) exhibited early leaf senescence with accumulation of hydrogen peroxide and reduced chlorophyll content (Han et al., 2014). The Stay-Green Rice (SGR) gene, encoding a chloroplast protein, is necessary for the initiation of chlorophyll breakdown (Park et al., 2007; Hörtensteiner, 2009) and it’s up-regulated expression induced leaf senescence (Ren et al., 2007; Pilkington et al., 2012). In addition, Rapid Leaf Senescence 3 (*RLS3*), which produces a protein with an AAAt domain, functions in delaying leaf senescence in rice (Lin et al., 2016). Mutation of *DEL1* (*Early Senescence Leaf 1*) decreases the enzymatic activity of PEL (pectate lyase) and increases expressions of *SAGs* (Leng et al., 2017). Although, various kinds of genes have been studied in rice, further investigation on leaf senescence-related genes is essential in order to establish a better understanding of regulatory mechanisms of senescence.

Glycosylation, a process of glycosyltransferases (GTs, EC 2.4.x.y) catalyzing the transfer of sugar moieties from activated donor molecules to specific acceptor molecules, is considered a single modification reaction on plant hormones, secondary metabolites, and xenobiotics by glycosidic bonds (Jones and Vogt, 2001; Lairson et al., 2008; Li et al., 2015). There are about 452 and 609 GT members in the *Arabidopsis* and rice genomes, respectively, and most of them have not been functionally characterized (Ko et al., 2006; Cao et al., 2008). UDP-glycosyltransferases (UGTs) utilize UDP-glucoses as donor in regulating various biological processes (Coutinho et al., 2003). Accumulating evidence suggests a critical role of UGTs in plant developmental processes and stress reactions. Reduced expression level of gene *UGT71B6* in *Arabidopsis* induces early senescence and enhances susceptibility to the necrotrophic pathogen *Alternaria brassicicola* (von Saint Paul et al., 2012). *UGT74E2* modulates plant architecture as well as conferring drought stress tolerance (Tognetti et al., 2010), and *UGT76C2* was found to be involved in adaptation to drought stress (Li et al., 2015). *UGT75D1* modulates cotyledon development and stress tolerance during seed germination (Zhang et al., 2016). Ectopic expression of *UGT85A5* in tobacco and *SrUGT74G1* in *Arabidopsis* promotes seed germination in tobacco (Sun et al., 2013) and catechin accumulation in *Arabidopsis* (Guleria and Yadav, 2014), respectively. Overexpression of *UGT80B1* increases resistance to freezing and heat stress (Mishra et al., 2015). Ectopic expression of *UGT85U1*, *UGT85U2*, and *UGT85V1* in *Arabidopsis* improved salt and oxidative stress tolerance (Ahrazem et al., 2015). In rice, the expression level of *OsGT61-1* was significantly responsive to exogenous treatment of ABA and NaCl (Singh et al., 2010). In addition, *XAX1* from GT 61, could mediate xylosyl transfer to rice xylan (Chiniquy et al., 2012). GT43 family is involved in xylan biosynthesis of rice (Lee et al., 2014). *OsGT47A* has a role in plant secondary cell wall thickness (Zhang B. et al., 2014).

Although several *SAGs* have been cloned and studied, there is no insight about the role of GT in leaf senescence. In the previous study, we described *Premature Leaf Senescence* mutant *(pls2*) in rice, mapped the *PLS2* locus on chromosome 3, and postulated the *LOC_Os3g15840*, a glycosyltransferase encoding gene, as the candidate gene (Zhang T. et al., 2014). In the present study, we remapped*PLS2* using a newly developed genetic population, and confirmed *LOC_Os3g15840* as the target gene. *PLS2* expression was detected in all tissue types, but predominantly in leaf mesophyll cells, with a sub-cellular localization of endoplasmic reticulum. The mutation in the *PLS2* caused PLS. Compared to the wild type (WT), the *pls2* mutant accumulates sucrose together with decreased expression of sucrose metabolizing related genes. The collected data suggest that *PLS2* is essential for normal senescence.

## Materials and Methods

### Plant Materials and Growth Conditions

Rice premature leaf senescence *pls2* was obtained as a space-radiation mutant in *indica* var. Luhui H103. The *pls2* mutant was crossed with *japonica* var. Nipponbare and a F_2_mapping population was grown in a paddy field in Beijing (39°54′N, summer season, temperate climate).

### Quantitative Analysis of Chlorophyll Content

Chlorophyll content was measured according to the procedure described by Suzuki and Makino (2012). About 0.2 g of leaves were homogenized in 5 ml of a 9: 1 acetone to 0.1 M NH_4_OH solution and centrifuged at 3000 × *g* for 20 min. These supernatants were then washed three times using hexane (1:1 ration of supernatants to hexane) and the pigment content was measured by spectrophotometer at the absorption wavelengths of 663 and 645 nm (Beckman Coulter DU-800, CITY, United States). The experiment was carried out with three technical and three biological replicates, respectively.

### Analysis of H_2_O_2_, Malondialdehyde (MDA)

H_2_O_2_ was detected by DAB staining as described by Thordal-Christensen et al. (1997). Fully expanded flag leaves were vacuum-infiltrated with DAB solution (1 mg of DAB dissolved in 1 ml of distilled water, pH 3.8) for 24 h at 25°C and washed in boiling ethanol (96%) for 10 min before photographing. For H_2_O_2_ quantitative measurement, H_2_O_2_ was extracted from leaves at heading according to the method described by Rao et al. (2000). MDA content was measured by the following steps: 0.5 g leaves were ground into powder, and then dipped into 0.5% TCA buffer, followed by treatment of 100°C for 30 min. After centrifuged at 3000 × *g* for 30 min, the supernatant (2 ml) was added to 2 ml 0.5% TBA, then subjected to 100°C for 30 min again. After the mixture was dropped to room temperature, it was centrifuged at 3000 × *g* for 20 min. Finally, the light absorption of the supernatant was measured at the absorption wavelength of 450-, 532-, and 600 nm, respectively. The MDA content was calculated according to the formula: (C_MDA_ = 6.45^∗^[A_532_-A_600_]-0.56^∗^A_450_ [μmol/L]). The experiment of H_2_O_2_ and MDA were performed with three technical and three biological replicates.

### Analysis of Photosynthetic Parameters

Flag leaves of the wild type and *pls2* mutant were used to measure the net photosynthetic rate, stomatal conductance, and transpiration rate from 9:00 am to 11:00 am. The detailed methods of photosynthetic parameters were to procedures (Wang et al., 2015). In order to allow flag leaves to reach steady-state photosynthesis, flag leaves were kept under each level of CO_2_ concentration for 5 min before these photosynthetic parameters were recorded on portable photosynthetic system (CIRAS-2, PP Systems, Hitchin, United Kingdom). The assay was carried out with three technical and biological replicates, respectively.

### Transmission Electron Microscopy (TEM)

Leaves at heading were cut into small pieces, fixed in 2.5% glutaraldehyde in a phosphate buffer (pH 7.2), vacuum infiltrated, rinsed, and incubated overnight at 4°C in a solution of 1% OsO_4_. Samples were dehydrated in a series of 10, 30, 50, 70, 90, and 100% ethanol and infiltrated in epoxy resin, and embedded in Epon 812 resin. A series of 80 nm sections was cut using a Reichert OM2 ultramicrotome, stained in 2% uranylacetate and 10 mM lead citrate (pH12), before observation in a HitachiH-7650 transmission electron microscope.

### Map-Based Cloning of the *PLS2* Gene

DNA was extracted according to CTAB method described by Telzur et al. (1999). Eight-hundred-and twenty *pls2*-like individuals were sampled from the segregating F_2_ population for linkage analysis. All InDel markers used in this study were developed according to sequence diversity between H103 and Nipponbare, which are available at the Gramene website^1^. dCAPS markers were automatically designed using the web server program dCAPS Finder 2.0.^2^ The *PLS2* locus was finally mapped to a 90-Kb region of chromosome 3 delimited by two dCAPS of C-2 and SL-1-9. The candidate gene was identified by DNA sequencing.

### Vector Construction and Transformation

For overexpression of *PLS2*, we cloned the coding region of *PLS2* into the pCAMBIA1390 vector under the maize *Ubi* promoter to produce the fusion vector *pUbi*::*PLS2*. The *pUbi*::*PLS2* vector was transformed into *pls2* plants by *Agrobacterium*-mediated transformation as described previously by Hiei and Komari (2008).

To obtain a crispr-*PLS2* mutant line, we used the CRISPR-Cas9 system according to the method previously described by Miao et al. (2013). A 20 bp *PLS2*-specific spacer sequence was cloned into the entry vector pOs-sgRNA, followed by subcloning into a pCAS9 binary vector by means of the Gateway cloning system. The fused vector was transformed into Nipponbare as described above. The molecular markers used for vector construction are listed in **Supplementary Table S1**.

### Quantitative Real-Time PCR Analysis

Total RNA was extracted according to the instructions with TRIZOL Kit (TaKaRa, Japan). First-strand cDNA was obtained from 2 μg of total RNA using the QuantiTect Reverse Transcription Kit (Qiagen, Germany). qRT-PCR (20 μl reaction volume) was carried out with 0.5 μl of cDNA, 0.2 μM of primer mix, and the SYBR Premix Ex Taq Kit (TaKaRa, Japan). The endogenous rice *UBQ* gene (*LOC_Os03g13170*) and *OsActin* gene (*LOC_Os03g50885*) were used as the reference genes, respectively. The assay was carried out with three technical and biological replicates respectively. All qRT-PCR primers are listed in the **Supplementary Table S1**.

### RNA *in Situ* Hybridization

Assays were performed as described previously (Bradley et al., 1993). For preparation of materials, flag leaves of wild type plants at heading were fixed using an RNase-free formalin/acetic acid fixative solution, followed by a series of dehydration steps and embedded in paraffin for sectioning. To prepare the probe, we used a pair of primers, PLS2-*in situ*-F (5′-CGTCAGTAGCTATTGCCGAGGACTTTGA-3′) and PLS2-*in situ*-R (5′-GCTTGTGAGAGCTCCTCGCCTT-3′), to amplify a 335 bp unique sequence of PLS2 from a cDNA clone. The fragment was then inserted into the pGEM-T vector (Promega) for RNA transcription. Digoxigenin-labeled RNA probes were prepared using a DIG Northern Starter Kit (Roche^3^). Hybridization signals were visualized and photographed using a Leica DMR microscope equipped with a Micro Color charge-coupled device camera (Apogee Instruments^4^).

### Subcellular Localization and Promoter Fusions

To create the integrated vector pCAMBIA1305-d35S-PLS2-GFP, the *PLS2* CDS fragment was cloned into the pCAMBIA1305-GFP vector at the *Bgl* II site. Constructs were transiently expressed in tobacco (*Nicotiana benthamiana*) epidermal cells as described previously (Batoko et al., 2000). Tobacco leaf protoplasts were obtained as described by Miao et al. (2006) to analyze transient expression of *PLS2*. The GFP signal was photographed by a laser scanning confocal microscope (LSCM 700; Carl Zeiss).

A 3,393 bp upstream fragment of the *PLS2* gene was amplified using the primers (**Supplementary Table S1**) and sub-cloned into a pCAMBIA 1305:GUS vector with restriction enzyme sites *EcoR* I and *Noc* I to get a *PLS2_Pro_*: GUS construct, and then introduced into *japonica* var. Kitaake by *Agrobacterium* described above. For histochemical analysis, we used excised tissues of independent T_2_ transgenic plants, as reported by Jefferson (1987).

### Quantitative Analysis of Soluble Sugar

Flag leaf pieces (0.05 g) were dissolved in 1 ml of 80% ethanol, incubated at 50°C for about 30 min, and then centrifuged at 3,000 *g* for 15 min, repeated twice. The three supernatants were vacuum dried at 45°C, dissolved in 50°C preheated ultrapure water, and subjected to a 5 μm C18 extraction column (150 × 4.6 mm; Agilent Zorbax) for removing impurities. After that, the soluble sugar of supernatants was analyzed by High Performance Liquid Chromatography instrument (Shimadzu Company, Japan). The standard curve of sucrose was previously made including concentrations of 10 mg/mL, 5 mg/mL, 2.5 mg/mL, 1.25 mg/mL, and 0.625 mg/mL. The chromatography analysis and sample recovery steps were carried out according to the method described by Brushwood (1997). The assay was conducted with three technical and biological replicates, respectively.

## Results

### *pls2* Is a Premature Leaf Senescence Mutant

**FIGURE 1:**
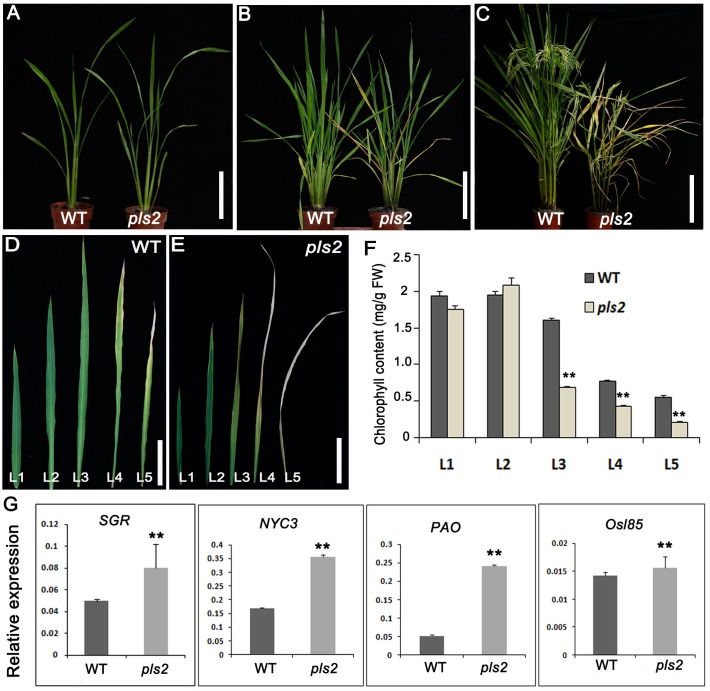
*pls2* is a premature leaf senescence mutant. **(A–D)** Phenotypes of wild type and *pls2* mutant at the seedling **(A)**, tillering **(B)**, and heading **(C)** stages. Bar in **(A–C)** 15 cm. **(D,E)** Uppermost five leaves from the main culms of WT **(D)** and *pls2* **(E)**. Bar in **(D,E)** 3 cm. L1–L5 represent leaves from the flag leaf downward. **(F)** Chlorophyll contents of the five uppermost leaves from WT and *pls2*. **(G)** qRT-PCR analysis of four senescence associated genes (*SAGs*) between WT and *pls2*. Data is presented as the mean ± standard deviation (*n* = 9). ^∗∗^*P* ≤ 0.01; Student’s *t*-test.

The senescence symptoms of the *pls2* mutant gradually developed from the tillering stage when emerging leaves slowly became yellowish or developed senescence (**Figure 1**). Senescent leaves had reduced chlorophyll content (**Figures 1D–F**). The *pls2* mutant had reduced height and internode length, and low seed setting (**Supplementary Figures S1A–C**). Detailed analysis of grain plumpness at different positions on the panicle revealed that the mutant had a higher proportion of semi-filled grain (SFGN), and shriveled grain (SGN) compared to WT (**Supplementary Figures S1D,E**). 1000-grain weight was decreased slightly, but not significantly in the *pls2* mutant (**Supplementary Figure S1F**). The results indicated that the PLS in *pls2* had a detrimental effect on vegetative growth as well as grain development (**Supplementary Figure S1**). In addition, expressions of several *SAG* genes (*PAO*, *SGR*, *NYC3*, and *Osl85*) were also significantly increased in the *pls2* mutant at heading stage in comparison with WT (**Figure 1G**).

### The *pls2* Mutant Accumulated H_2_O_2_ and Reduced Leaf Photosynthetic Capability

**FIGURE 2:**
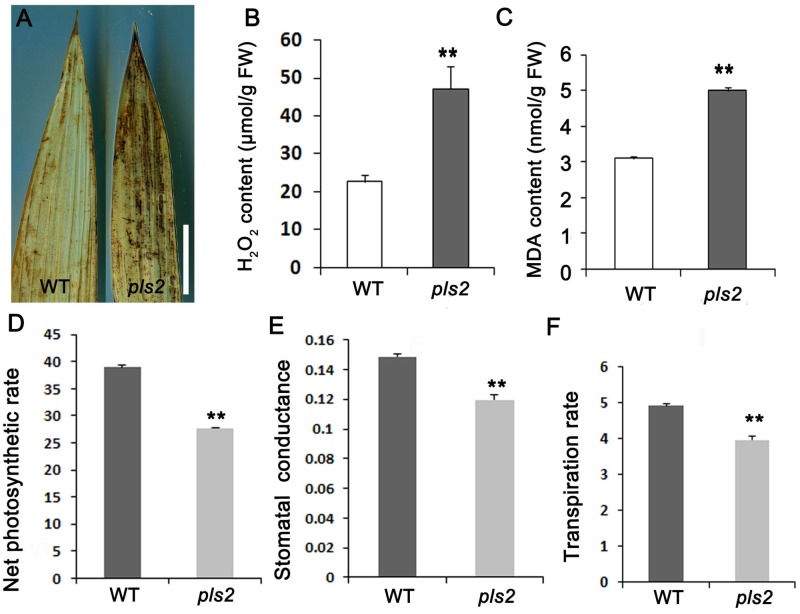
*pls2* accumulated excessive ROS. **(A)** DAB staining of leaves of WT and *pls2*. Bar, 4 cm. **(B,C)** H_2_O_2_ **(B)** and MDA **(C)** quantitation assays at heading. **(D–F)** Net photosynthetic, stomatal conductance, and transpiration rates in flag leaves of WT and *pls2*. Data is presented as the mean ± standard deviation (*n* = 9). ^∗^0.01 ≤*P* ≤ 0.05; ^∗∗^*P* ≤ 0.01; Student’s *t*-test.

We examined H_2_O_2_ [a reactive oxygen species (ROS)] levels by DAB in both WT and *pls2*, and strong DAB brown straining appeared in the *pls2* flag leaves (**Figure 2A**), consistent with quantitative results showing that the *pls2* mutant exhibited excess H_2_O_2_ accumulation (**Figure 2B**). As the end-product of membrane lipid peroxidation caused by ROS (Draper and Hadley, 1989), the malonic dialdehyde content (MDA) was accumulated highly in the *pls2* (**Figure 2C**). Meanwhile, leaf photosynthesis capability determined by net photosynthetic rate (**Figure 2D**), stomatal conductance (**Figure 2E**), and transpiration rate (**Figure 2F**) was accordingly reduced in *pls2.*

### Map-Based Cloning of the *PLS2* Gene

**FIGURE 3:**
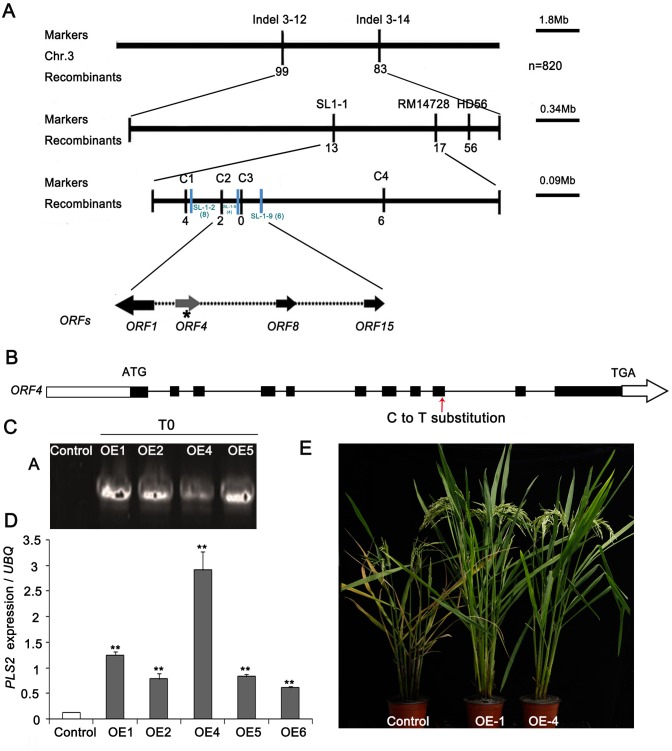
Map-based cloning of the *PLS2* gene. **(A)** Fine mapping of *PLS2* in the F_2_ population of *pls2* × Nipponbare. **(B)** Schematic of the *PLS2* gene structure. Black boxes indicate exons and black lines between the boxes represent introns. The red arrow indicated the position where the C→T substitution occurred. **(C,D)** Overexpression analyses of positive plants by qRT-PCR. **(E)** *PLS2* over-expressing plants in *pls2* background. Bar, 15 cm. ^∗∗^*P* ≤ 0.01; Student’s *t*-test. Data is presented as the mean ± standard deviation (*n* = 9).

Segregation in the redeveloped F_2_ population of *pls2* × *japonica* cv. Nipponbare was 2,285 plants with normal phenotype and 820 with mutant phenotype, confirming that a single recessive gene caused the senescent phenotype (χ^2^_3:1_ = 0.06: *P* > 0.05). We previously placed *PLS2* between markers of RM14704 and SL-1-5 on chromosome 3, represented by a physical distance of 84.11 Kb (Zhang T. et al., 2014). Using extra 820 F_2_ mutant individuals, we confirmed the interval by the newly developed marker C-2 and SL-1-9 (**Figure 3A**), which mapped the similar location compared with previous study. The determined 90 Kb candidate region contained 15 ORFs, and a C→T substitution in the ninth exon of gene LOC_Os03g15840 (ORF4), putatively causing an R→C amino acid alternation (**Figure 3B**).

To validate the candidate gene, we transformed the WT CDS of LOC_Os03g15840 driven by the *Ubi* promoter into the *pls2* mutant. Four independent positive T2 transgenic plants were identified by PCR genotyping (**Figure 3C**) and qRT-PCR, which expressed LOC_Os03g15840 significantly higher than the control (**Figure 3D**) and rescued the leaf senescence phenotype of the *pls2* mutant (**Figure 3E**), confirming that an aberrant LOC_Os03g15840 was the cause of *pls2*. We further verified the candidate gene by knock-out of the gene in Nipponbare background using Crispr/Cas9 technology. Finally, two Crispr-*PLS2* positive transgenic plants exhibited early leaf senescent and reduced plant height (**Supplementary Figure S2**). These results indicated that the mutation in LOC_Os03g15840 caused the leaf senescence of the *pls2*, and the locus therefore was designated *PLS2*.

LOC_Os03g15840 encodes a glycosyltransferase containing the Glyco_trans_4_1 and Glyco_trans_4_2 domains that catalyze sugar transfer from donor to acceptor (**Supplementary Figure S3A**). A phylogenetic tree analysis showed that the rice *PLS2* gene Os03g0265100 is highly homologous to *Brachypodium sylvaticum* gene XP003558286.1 and *Aegilops tauschii* gene EMT15626.1 (**Supplementary Figure S3B**).

### Temporal and Spatial Expression of the *PLS2* Gene

**FIGURE 4:**
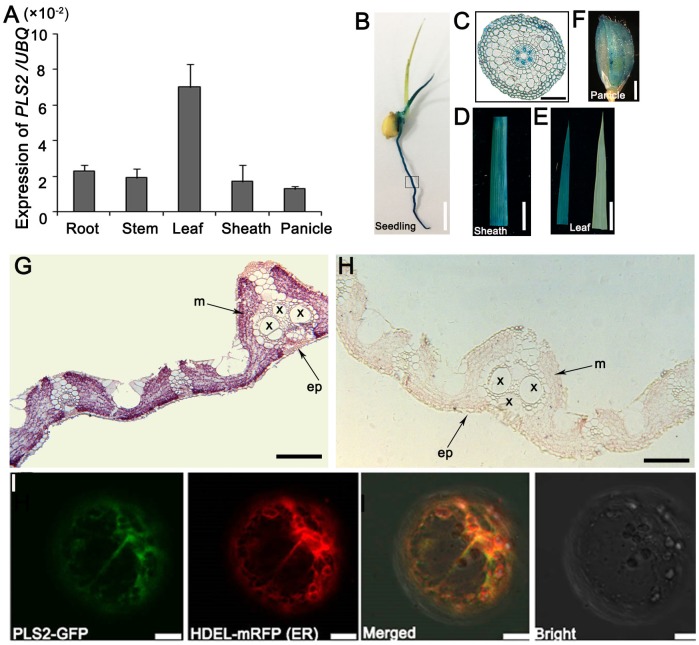
Expression and localization of PLS2. **(A)** Expression levels in various tissues revealed by qRT-PCR using the *UBQ* as the reference gene. Data is presented as the mean ± standard deviation (*n* = 9). **(B–E)** GUS expression patten of *P_PLS2_:GUS* transgenic rice plants on 5-day-old seedlings **(B)**, root cross-scection of the box area in B **(C)**, leaf sheath **(D)**, leaf (**E**; left, *P_PLS2_:GUS* transgenic leaf; right, control). **(G,H)** RNA *in situ* hybridization analysis of *PLS2*. Flag leaves of wild type plants at heading were cross-sectioned and hybridized with *PLS2*-specific antisense **(G)** or sense **(H)** probes. x, xylem; p, phloem; ep, epidermis; m, mesophyll. **(I)** Co-expression of PLS2-GFP fusion protein with HDEL-mRFP (ER marker). ER, endoplasmic reticulum. Scar bars: 12 mm in **(B)**, 100 μm in **(C)**, 1 cm in **(D,E)**, 3 mm in **(F)**, 200 μm in **(G,H)**, 10 μm in **(I)**.

The results of qRT-PCR showed that *PLS2* was expressed in all tissues, i.e., roots, leaf sheaths, stems, young leaves, and panicles, and there was strong expression in leaves, followed by roots and stems (**Figure 4A** and **Supplementary Figure S4**). *β*-glucuronidase (GUS) staining was detected in various tissues, especially seedlings (**Figures 4B,C**), leaf sheaths (**Figure 4D**), leaves (**Figure 4E**), and panicles (**Figure 4F**). Observations on root sections indicated strong GUS signals in the phloem (**Figure 4C**). *In situ* hybridization (ISH) assays demonstrated that *PLS2* was highly expressed in leaf mesophyll cells (**Figures 4G,H**). The PLS2-GFP completely merged with the ER marker HDEL-mRFP (**Figure I**) (Gomord et al., 1997) in epidermal protoplasts of *N. benthamiana* leaves, suggesting subcellular localization of the PLS2 in the endoplasmic reticulum (ER).

### *pls2* Accumulated More Sucrose Than WT

**FIGURE 5:**
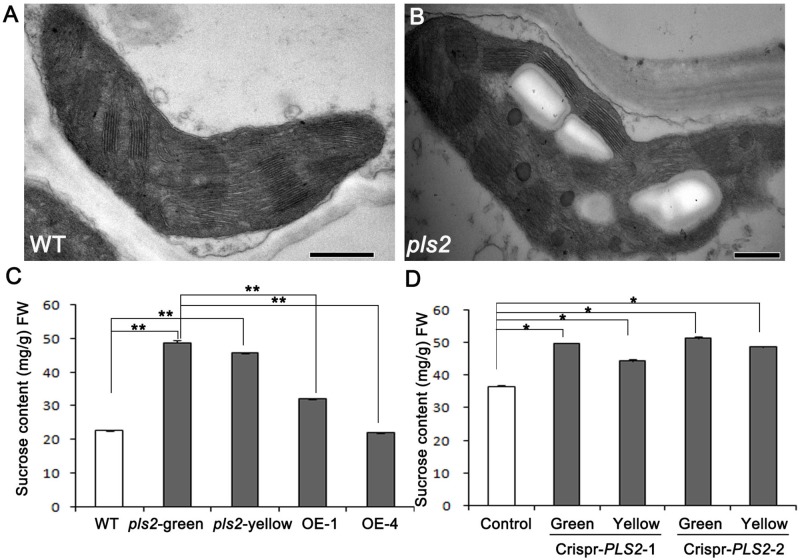
Sucrose contents of plants at heading. **(A,B)** Transmission electron microscopic images of flag leaf cells from wild type **(A)** and *pls2* mutant **(B)**. Bar 500 nm in **(A,B)**. **(C)** Sucrose content in WT, *pls2*, and OE lines of OE1 and OE4. **(D)** Sucrose content in the control and two crispr plants. Data is presented as the mean ± standard deviation (*n* = 9). ^∗^0.01 ≤*P* ≤ 0.05; ^∗∗^*P* ≤ 0.01; student’s *t*-test.

Through TEM assay, we found that cells in green sections of *pls2* leaves were heteroplastidic with more starch grains (**Figures 5A,B**). It is well known that glycosyltransferases catalyze the transfer of activated sugars to various acceptor molecules (Li et al., 2015; Zhang et al., 2016). In order to investigate the impact of *PLS2* mutation on the sugar metabolism, sucrose levels in flag leaves at grain filling stage were measured in the WT, *pls2*, *Ubi*::*PLS2*-OE, and CRISPR/Cas9-based knockout-plants. Results revealed that the *pls2* accumulated more sucrose than WT whether in their green leaves or senesced leaves (**Figure 5C**). Transgenic over-expressing lines showed similar sugar levels to WT (**Figure 5C**). Two CRISPR knockout plants accumulated excess sucrose compared to the control (**Figure 5D**).

### Expression Level Analysis of Sucrose-Related Genes

**FIGURE 6:**
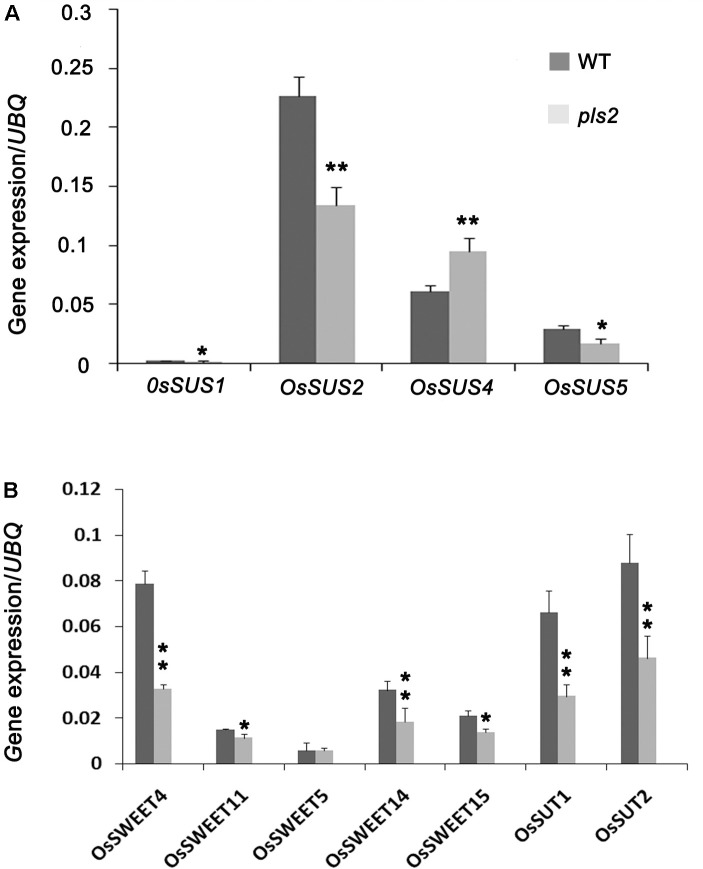
Expression analyses of genes associated with sucrose synthesis genes *OsSUS1*, *2*, *4*, *5* **(A)** and sucrose transportation genes *OsSWEET4*, *5*, *11*, *14* and *OsSUT1*, *2* **(B)**. Data is presented as the mean ± standard deviation (*n* = 9). ^∗^0.01 ≤*P* ≤ 0.05, ^∗∗^*P* ≤ 0.01, Student’s *t*-test.

Sucrose synthase (SuSy, EC 2.4.1.13) mainly mediates the sucrose metabolism by catalyzing the reversible transfer of a glucosyl moiety between fructose and a nucleoside diphosphate (NDP) (Chourey et al., 1998; Baroja-Fernández, 2012). There are at least six members (OsSUS1-6) of sucrose synthase family in rice (Tatsuro et al., 2008). We assayed the expression levels of sucrose synthase genes *OsSUS1*, *OsSUS2*, *OsSUS4*, and *OsSUS5* (Hirose et al., 2008). qRT-PCR analysis showed that mRNA levels of all except *OsSUS4* were remarkably down-regulated in *pls2* (**Figure 6A**), suggesting that lower expression suppresses sucrose turnover and metabolism, and result in sucrose accumulation in *pls2*. We next detected the expression level of sugar transporters including *OsSUT1* (Siahpoosh et al., 2012), *OsSUT2* (Siao et al., 2011), *OsSWEET4*, *OsSWEET5* (Zhou et al., 2014), *OsSWEET11*, *OsSWEET14* and *OsSWEET15* in WT and the *pls2* mutant, respectively, and found that all mRNA levels were significantly down-regulated in *pls2* (**Figure 6B**), suggesting that mutation in the *PLS2* gene affected the sugar transport, leading to over-accumulation of starch in chloroplast of *pls2*.

## Discussion

Premature leaf senescence causes detrimental plant growth and reduced crop productivity (Chen L.J. et al., 2013; Chen Y. et al., 2013; Liang et al., 2014). Previous studies on leaf senescence revealed that many genes are involved (Woo et al., 2001; Liang et al., 2014; Schippers et al., 2015) and *SAGs* usually up-regulated during senescence (Liang et al., 2014; Sakuraba et al., 2015; Yang et al., 2015; Zhao et al., 2015, 2016; Zhu et al., 2015). Our study found that several *SAGs* such as the pheophorbidea oxygenase gene *PAO* (Schelbert et al., 2009), chloroplast degradation related gene (*SGR*) (Hörtensteiner, 2009), and senescence associated genes *NYC3* and *Osl85* (Lee et al., 2001), were prominently up-regulated in the *pls2* mutant, suggesting *pls2* was undergoing a typical senescence process. However, identification of more SAGs could be helpful in elucidating the leaf senescence processes involved in normal plant development. Here, we characterized a rice *pls2* mutant that displayed PLS under normal conditions and isolated a putative glycosyltransferase encoding gene LOC_Os03g15840by map-based cloning. A mutation in *PLS2* was responsible for the defective phenotype. *PLS2* expression was detected in all tissues surveyed, but predominantly in leaf mesophyll cells, with a sub-cellular localization of the endoplasmic reticulum. The *pls2* mutant accumulated much higher levels of sucrose together with decreased expression of sucrose metabolizing related genes compared to wild type. Our results indicated that the *PLS2* is essential for normal leaf senescence and its mutation resulted in the PLS.

Based on the substrate specificity of GT, all GT members can be classified into 105 GT subfamilies in *Arabidopsis* and 41 OsGT in rice (Ko et al., 2006; Cao et al., 2008). UDP glycosyltransferases are the most common GT enzymes that catalyze glycosylation in the plant kingdom. They transfer donor molecules to specific acceptors and participate in adversities with environmental conditions (Coutinho et al., 2003; Lairson et al., 2008). A recent study shows that excessive UGT results in programmed cell death (PCD) (Xiao et al., 2018). However, information on GT function in PLS is scarce. According to the gene annotation, *PLS2* is an unidentified substrate GT and our results provide evidence that abnormal sugar metabolism leads to leaf senescence.

Reactive oxygen species are continuously produced in plants as products of aerobic metabolism (Maurino and Flügge, 2008). Excess ROS accumulation leads to oxidative damage to thylakoid membranes and other cellular components (Apel and Hirt, 2004). Previous studies showed that early leaf senescence is usually associated with excessive ROS (Han et al., 2014). In our study, one species of ROS, H_2_O_2_, was much higher in the *pls2* mutant than wild type, indicating that H_2_O_2_ accumulation in *pls2* might result in oxidative impair to thylakoid membranes, and finally causing PLS in the mutant. Previous studies suggested that glycosyltransferases in rice and *Arabidopsis* were mostly localized in cytoplasm (Dong et al., 2014; Liu et al., 2015). Our investigation with marker HDEL-mRFP (Gomord et al., 1997) indicated PLS2 was completely merged with ER in epidermal protoplasts of *N. benthamiana* leaves, suggesting that PLS2 perform its role in the ER.

Sugars, especially sucrose, are not only essential carbon and energy sources, but also exert regulatory roles in metabolism control, stress immunity, growth, and development (Rolland et al., 2006, a review). Previous studies showed that exogenous sucrose supplied to leaves affects sugar metabolism and inhibits photosynthesis by down-regulating Rubisco abundance activity in 4-month-old sugarcane (Lobo et al., 2015). Feeding glucose or sucrose to mesophyll protoplasts in basic maize medium decreased photosynthetic gene expression (Sheen, 1990). In addition, leaf senescence was induced by adding glucose in combination with low nitrogen levels in *Arabidopsis* (Pourtau et al., 2004; Wingler et al., 2004). Over-accumulation of sugar accelerated the synthesis of starch grains in chloroplast stroma, which resulted in oppressing thylakoid and inhibiting light absorption in photosynthetic membranes (?). Here, we found that *PLS2* mutation caused significant sucrose accumulation and cells containing chloroplasts were heteroplastidic with starch grain accumulation in *pls2*, speculating that larger numbers of starch grains in chloroplasts and the descending expression of photosynthesis genes might cause the premature leaf senescence in *pls2*.

Sucrose is the main form of photosynthetic product and its translocation and distribution are mainly regulated by SUTs. SUTs are mainly involved in phloem loading, long-distance transportation, “library” unloading of sucrose (Kühn et al., 2003; Hackel et al., 2006), and regulating sucrose storage and distribution (Rae et al., 2005; Hackel et al., 2006). One study showed that *OsSUT1* expression is inhibited by higher sucrose content and induced by drought and salt stress in excised rice tissues (Ibraheem et al., 2011). An *Ossut2* mutant showed sucrose accumulation and lower sucrose output capability that finally disturbed plant development (Eom et al., 2011). When excised tobacco (Krapp et al., 1991) and barley (Parrott et al., 2005) leaves were exposed to strong sunlight more sugar accumulated and caused PLS. The *pls2* mutant similarly undergoes sugar accumulation that appears to be the cause of PLS. However, due to lack of knowledge of the substrate of the glycosyltransferase encoded by *PLS2* and its interacting protein, the mechanism of senescence underlying *pls2* remains to be explored.

## Author Contributions

MW and ZC designed the experiment. MW, TZ, and HP performed most of the experiments. SL, JT, KJ, YH, XZ, and XG participated in some part of the study. MW wrote the paper. JZ and ZC edited the manuscript.

## Conflict of Interest Statement

All The authors declare that the research was conducted in the absence of any commercial or financial relationships that could be construed as a potential conflict of interest.

## Footnotes

1 www.gramene.org

2 http://helix.wustl.edu/dcaps/dcaps.html

3 http://www.roche.com/

4 http://www.apogeeinstruments.com/

## Supplementary Material

FIGURE S1 Agronomic trait comparisons between the wild type (WT) and *pls2* in plant height **(A)**, and internode lengths **(B)**, seed setting **(C)**, three types of grain **(D)**, their proportions **(E)**, and 1000-grain weight **(F)**. FGN, fully filled grain; SFGN, semi-filled grain; SGN, shriveled grain, bar, 5 mm. Data is presented as the mean ± standard deviation (*n* = 9). ^∗^0.01 ≤*P* ≤ 0.05; ^∗∗^*P* ≤ 0.01; Student’s *t*-test.

FIGURE S2 *PLS2* crispr analyses. **(A)** Crispr plants in Nipponbare background. Bar, 15 cm. **(B)** Plant height of crispr plants with the control. **(C)** Sequence analysis of *PLS2* for Crispr lines. ^∗∗^*P* ≤ 0.01; student’s *t*-test. Data is presented as the mean ± standard deviation (*n* = 9).

FIGURE S3 Phylogenetic analysis of PLS2 protein and homologous proteins among plant species. **(A)** The domains of PLS2 protein. **(B)** Phylogenetic and blast analyses of PLS2 among plant species.

FIGURE S4 Expression levels in various tissues revealed by qRT-PCR using the *OsAction* as the reference gene. Data is presented as the mean ± standard deviation (*n* = 9).

TABLE S1 Primers used in the study.
