# Supplementary figures and images for: Rice Premature Leaf Senescence 2, Encoding a Glycosyltransferase (GT), Is Involved in Leaf Senescence
Source: Front Plant Sci. 2018 Apr 26;9:560. doi: 10.3389/fpls.2018.00560 (PMC5932172; doi:10.3389/fpls.2018.00560)

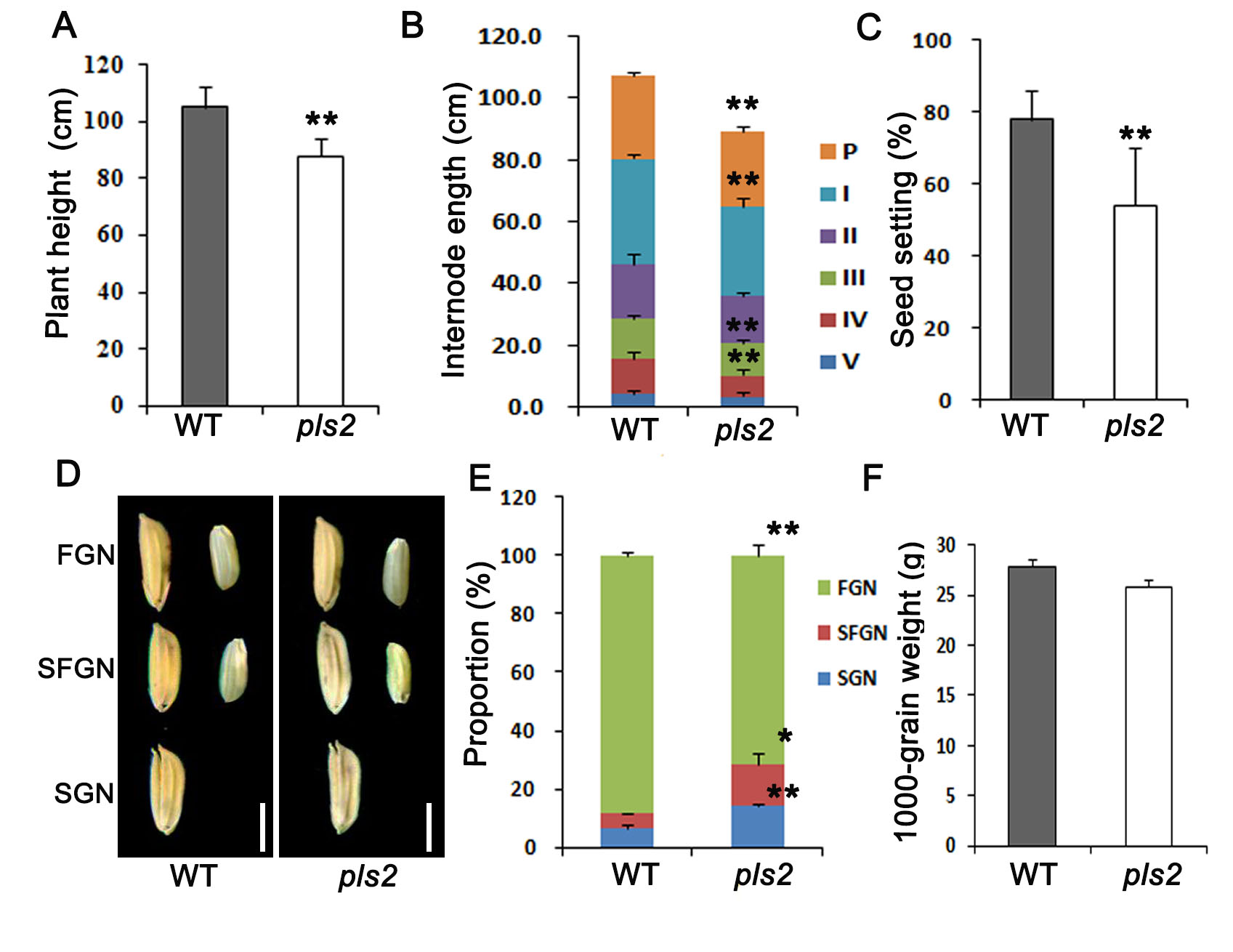

Supplement: FIGURE S1 — Agronomic trait comparisons between the wild type (WT) and pls2 in plant height (A), and internode lengths (B), seed setting (C), three types of grain (D), their proportions (E), and 1000-grain weight (F). FGN, fully filled grain; SFGN, semi-filled grain; SGN, shriveled grain, bar, 5 mm. Data is presented as the mean ± standard deviation (n = 9). ∗0.01 ≤P ≤ 0.05; ∗∗P ≤ 0.01; Student’s t-test. [file Image_1.JPEG]

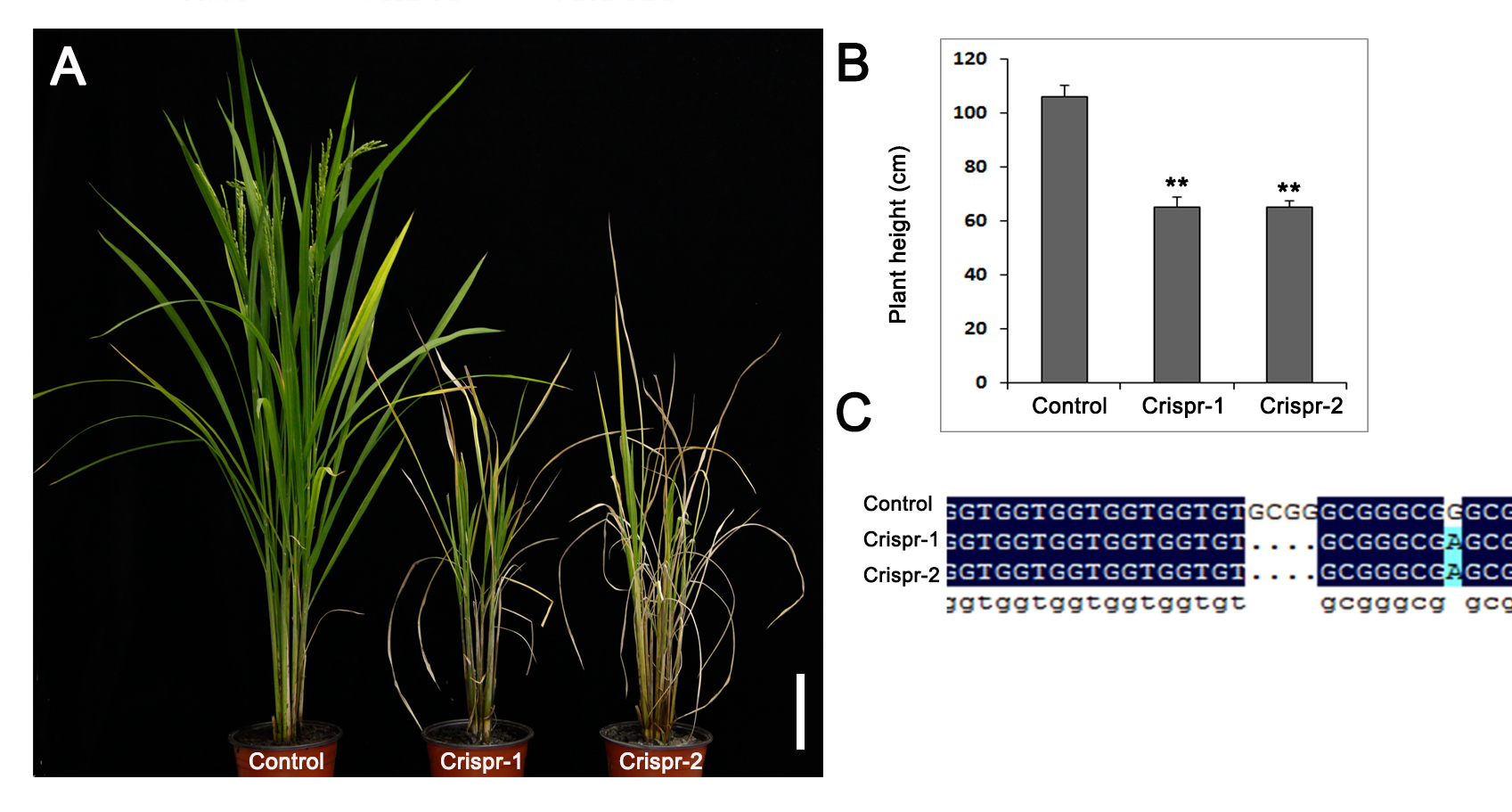

Supplement: FIGURE S2 — PLS2 crispr analyses. (A) Crispr plants in Nipponbare background. Bar, 15 cm. (B) Plant height of crispr plants with the control. (C) Sequence analysis of PLS2 for Crispr lines. ∗∗P ≤ 0.01; student’s t-test. Data is presented as the mean ± standard deviation (n = 9). [file Image_2.JPEG]

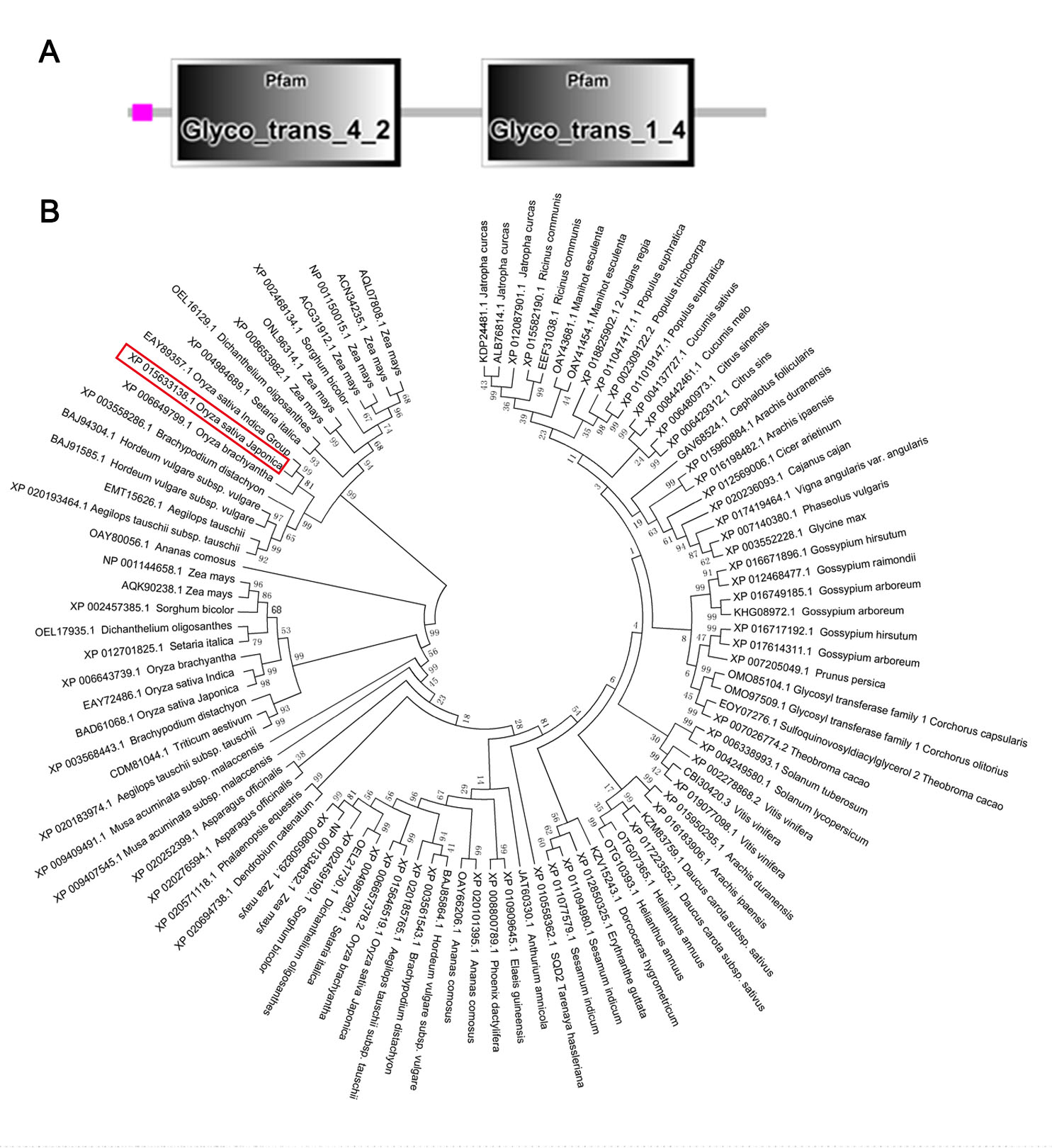

Supplement: FIGURE S3 — Phylogenetic analysis of PLS2 protein and homologous proteins among plant species. (A) The domains of PLS2 protein. (B) Phylogenetic and blast analyses of PLS2 among plant species. [file Image_3.JPEG]

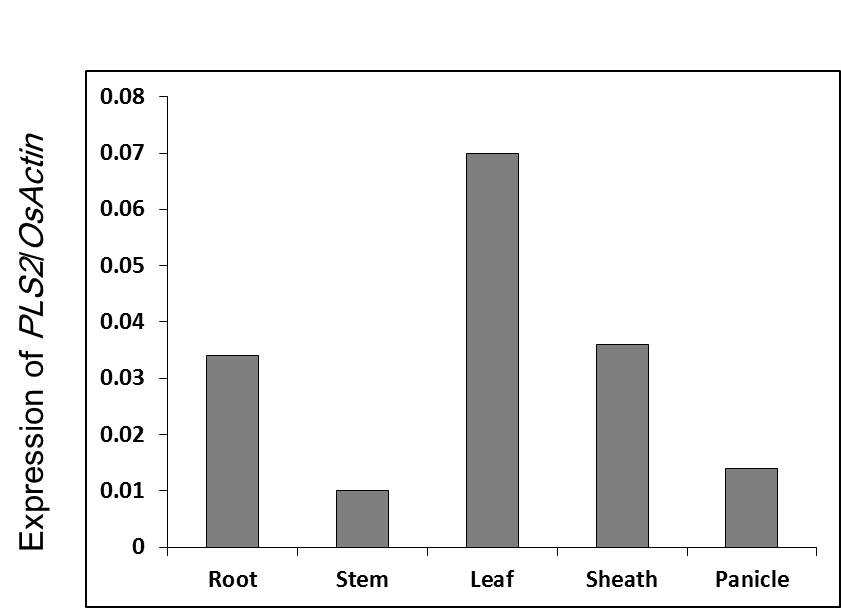

Supplement: FIGURE S4 — Expression levels in various tissues revealed by qRT-PCR using the OsAction as the reference gene. Data is presented as the mean ± standard deviation (n = 9). [file Image_4.JPEG]
