# Supplementary material for: Rice Premature Leaf Senescence 2, Encoding a Glycosyltransferase (GT), Is Involved in Leaf Senescence
Source: Front Plant Sci. 2018 Apr 26;9:560. doi: 10.3389/fpls.2018.00560 (PMC5932172; doi:10.3389/fpls.2018.00560)
Supplement: TABLE S1 — Primers used in the study. [file Table_1.PDF]

Table S1 Primers used in the study

Primers	Forward sequence (5'-3')	Reverse sequence (5'-3')
InDel3-12	CCAGGGATCTTCTCATCCAA	CCTGGCTAGCATACCACACA
InDel3-14	TATAGCGGACTGGCCAAACT	CCACCCATGTCATCTTCCAT
RM14704	GCTCAGCCAGCACACTTACG	TTCCTTCAGCATTCCAGTCTGC
RM14728	CTCAGCGGACTAGAATGCAAGC	AAACGGATACATCTTCGCAGAACG
HD56	GCCAGTCAAGTGACCAAACA	CACATCACGTGTACAGGTTCG
SL-I-1	TGACCAACAGTTCCCCAAAT	TCCCAATCTCCAATCAGAGC
SL-I-2	CGTCTTGCAGGCATTTCAGT	CTGATGATCCGGACCAATTT
SL-I-4	TCGTCCTTCAGATCCATTCG	CGGTTGATAGTGATGCCAAG
SL-I-5	AGGGGCCCTTAGTGAATCCT	GCTGCTCATGCTGTGTGTTT
SL-I-9	ATAACTTTGTCTGTAGCATGTA	ATGTCGCTGCCACAGTATCC
SL-I-10	AGATCCCATGAAAACCCCACTAAAA	AAGCCACACAACCTAACATGACAAC
dC-1	AATAACGATATGATGAATTTTTTTA	AGTTGAGATAGAATCGAAATTTC
d-2	TTCCAAAAGAACCTATCCATAAGCT	TAGCTTGAGGAGCTCAGCTTAATAC
dC-3	GGACGGGGAAGGGGGCGGGGAAGTC	GCGGCTGCCACCCTTGCCCTCTACT
dC-4	GACCGATGAGATCACGCGCTCGGGG	TTCTTCGGCGTCTTCATGGCCA
PLS2-1390-*Pst* I-Flg	TCTGCACTAGGTACCTGCAGATGGGGCAGCAGGCGG	ATGGATCCGTCGACCTGCAGCTAGTTAGTTCTGCCCATTT
1305-GFP-15840-*Bgl II*	CGAGCTGTACAGATCTATGGGGCAGCAGGCGGCG	GGCCGCTTTAAGATCTGTTAGTTCTGCCCATTTTCTT
2300-PLS2-*Sac* I	ATGATTACGAATTCGAGCTC TTGTATGAGCGAATGAGGGAA	ATGCCTGCAGGTCGAC TAGACAGCATTCTCAGTCAAGGTG
PLS2-in-situ	CGTCAGTAGCTATTGCCGAGGACTTTGA	GCTTGTGAGAGCTCCTCGCCTT
PLS2-1305-GUS	CCATGATTACGAATTCTTGTATGAGCGAATGAGGGAA	CTCAGATCTACCATGGCTCCTCCTCTGGCCCGCGTCG
PLS2-CRISPR	AGATGATCCGTGGCA GCGCAGCCGCTACTGCTGCA GTTTTAGAGCTATGC	GCATAGCTCTAAAACTGCAGCAGTAGCGGCTGCGCTGCCACGGATCATCT
SGR	AGGGGTGGTACAACAAGCTG	GCTCCTTGCGGAAGATGTAG
NYC3	TCTATCTAGGTGCCAAAGGC	ATTCTGGCACCTGCTGTTTC
PAO	AAGCCTCCGATGTTACCGAA	CGAGGGTTTCCAGAATTTGA
Osl85	GAGCAACGGCGTGGAGA	GCGGCGGTAGAGGAGATG
OsSUS1	GGAGGGCAGGTTGTCTACAT	TGACAATAAGGATGCGTGGT
OsSUS2	ACCTGATCTCATCATTGGCA	TGAGCAATGGTACACTGGGT
OsSUS4	AGCATTCGGTCTCACTGTTG	TCAGATTTGCAGCTTGATCC
OsSUS5	AAAGAGAAGCCTGGCCAATA	ACATTAATGCCTGTGGCGTA
OsSWEET4	ATCCGCTTCGACCTCTACAT	CTGCTGCGTCGACTTGTAGT
OsSWEET5	GCACCCTCTGCGTCTTCT	AGCATATGCCGTTGATGAAG
OsSWEET11	TCTCCTTCCTGGTGTTCCTT	TAGAAGATCCACAGCACCGA
OsSWEET14	ACCAAGAGCGTGGAGTTCAT	TGATGAGGAGGCCGTAGAG
OsSWEET15	GCGGTGATCTGGTTCTTGTA	CACCAGTGGCTTCTTGCTC
OsSUT1	TGGCCTTACCTGGTTGTCTTG	GGGTCACCGTGGTAGATCTCA
OsSUT2	CAAGGAGGAGAGGTCACCGATA	TGCCTCTTATGAGTCGAATTGC
PLS2	TTTAAGCCGGACATAATCCA	ACATCACCATTGGGACTGAA
UBQ	GCTCCGTGGCGGTATCAT	CGGCAGTTGACAGCCCTAG
OsActin	ATGGCTGACGCCGAGGATATCC	TTAGAAGCATTTCCTGTGCACA
